# Practical, effective and safer: Placing traps above ground is an improved capture method for the critically endangered ngwayir (western ringtail possum; *Pseudocheirus occidentalis*)

**DOI:** 10.1017/awf.2024.31

**Published:** 2024-09-16

**Authors:** Sara Corsetti, Kaori Yokochi, Evan Webb, Arianna Urso, Roberta Bencini

**Affiliations:** 1 School of Agriculture and Environment, The University of Western Australia, 35 Stirling Hwy, Crawley, WA 6009, Australia; 2 School of Life and Environmental Sciences, Deakin University, Burwood, VIC 3125, Australia

**Keywords:** Animal welfare, stress, trapping, western ringtail possum, wildlife, wildlife capture

## Abstract

The capture of wild-living animals can provide valuable information that is critical in developing and implementing effective conservation actions. These capture procedures, however, often require direct handling of individuals by researchers, and conservationists should constantly seek to improve capture methods so that the impacts on animal welfare are minimised. The ngwayir (western ringtail possum; *Pseudocheirus occidentalis*) is a critically endangered arboreal marsupial in need of effective conservation. It is, however, not amenable to conventional trapping, leading to the use of methods such as nest robbing and tranquilisation using dart guns or pole syringes, which involve potentially serious animal welfare risks and longer exposure of animals to humans as compared to conventional trapping. In pursuit of an improved capture method, we investigated opportunistically whether placing traps above the ground would increase the capture success rate of the species, using wire cage traps baited with universal bait and fruit. Between 2010 and 2019, we deployed trapping grids in Locke Nature Reserve and adjacent campsites near Busselton, WA, Australia, with traps placed on the ground for 1,985 trap nights and traps placed on horizontal tree branches, fallen trees or fences, 1–2 m above ground for 694 trap nights. With the above ground traps we trapped 82 ngwayirs out of 694 trap nights, 27 in autumn and 55 in spring. We also captured eleven common brushtail possums (*Trichosurus vulpecula*; 1.6% trap success rate), 12 King’s skinks (*Egernia kingii*; 1.7%) and five black rats (*Rattus rattus*; 0.7%). Trapping success rate was higher in elevated traps (up to 18.3%) compared to traps on the ground (0.5%) and using fruit as bait increased the trap success rate. These results suggest that using elevated traps baited with fruit is a practical, effective method to capture the ngwayir.

## Introduction

Human actions and developments often negatively impact the welfare of wildlife both directly and indirectly (e.g. hunting, motor vehicle collisions, habitat destruction) and, for many wildlife species, accumulation of these impacts can place the entire population or species at risk of extinction (Hoffmann *et al.* 2011, Woinarski *et al.* 2014). Conservationists seek to reduce and ultimately mitigate these impacts and risks to wildlife. To develop and implement effective conservation actions, conservationists require extensive knowledge regarding the target community of wildlife and threats that operate on the community; however, obtaining such information often entails collecting data from wildlife, which could impact the welfare of individual animals (McMahon *et al.* 2012). For instance, the capture of wild-living animals can provide conservationists with valuable information on population demographics, health and reproductive status, movement, and/or survival of individual animals, which are critical in identifying key threats and developing and implementing effective solutions. Capture procedures, however, often require direct handling of individuals by researchers, and conservationists should constantly seek to improve capture methods so that the impacts on animal welfare are minimised (DelGiudice *et al*. 2005, Hampton & Arnemo 2023). The benefit of a novel, less-impactful capture method is even greater if it is effective and more time and cost-efficient than conventional methods because this will encourage its wider uptake.

**Figure 1.**
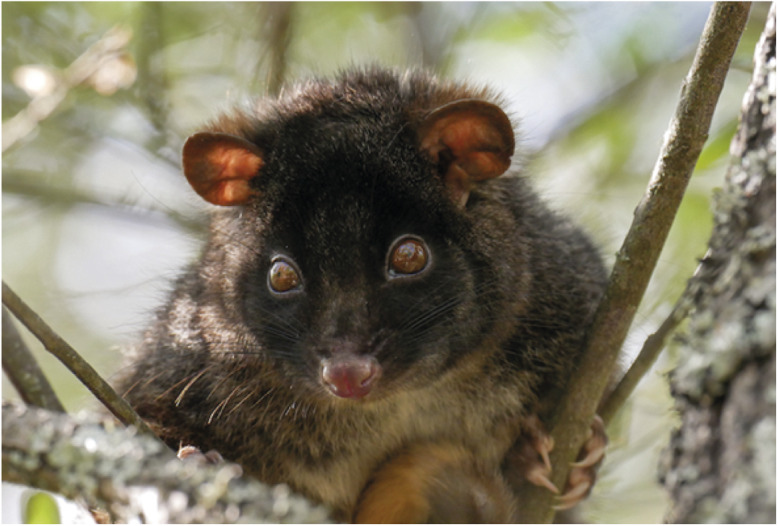
The ngwayir, or western ringtail possum (*Pseudocheirus occidentalis*), an arboreal marsupial endemic to southwest Western Australia, has recently been reclassified as critically endangered (Photograph: E Webb).

A species for which the development of a simpler, effective method of capture would be beneficial is the ngwayir, or western ringtail possum (*Pseudocheirus occidentalis*), an arboreal marsupial endemic to southwest Western Australia (Jones *et al.* 1994a,b). The species is listed as critically endangered at both state and federal government levels and is one of 20 mammalian species for which the Australian Government is prioritising recovery efforts (Burbidge & Zichy-Woinarski 2017; Department of the Environment 2021; Figure 1). As part of these efforts, a Recovery Plan has been developed for the species, which lists gaps in knowledge as a threatening process (Department of Parks and Wildlife 2017). Much of the current body of knowledge on the ngwayir originates from short-term studies, and knowledge gaps are exacerbated by difficulties in capturing the species related to their arboreal, folivorous nature (Department of Parks and Wildlife 2017).

To date, the capture-mark-recapture method has not been applied to estimate population sizes of the ngwayir because they are not considered amenable to conventional capture methods, such as trapping, and will not readily enter cage traps (Jones *et al.* 1994a; Grimm & de Tores 2009; de Tores & Elscot 2010; Zimmermann 2010). Given this, researchers used to rely upon manual capture of this nocturnal species by directly shepherding the animal to the ground using an extended hook at night or searching for their nests (dreys) during the day and capturing the animals by hand while they were asleep, a method known as ‘drey robbing’ (Jones *et al.* 1994b). These manual capture methods were stressful for both animals and researchers (P de Tores, personal communication 2010), which led to the development of labour-intensive methods to capture the species, involving spotlighting to search for individuals at night and immobilising them with a dart gun or with a pole syringe, typically using a chemical immobilisation regime with tiletamine-zolazepam (Hunter 2012; Clarke *et al.* 2013; Yokochi *et al.* 2016).

Although a chemical immobilisation regime might be less stressful for the animals because it ensures an alteration of consciousness with absence of awareness during the capture and handling of the animals (Bonhomme *et al.* 2019), administration of a chemical immobilisation regime using firearms entails risking hitting the target animal in the wrong place, such as the pouch, resulting in injury or death of pouch young, or the scrotum, resulting in injury. Although extremely rare (0.6%), these incidents have been witnessed (K Yokochi, personal communication 2015) and reported to the UWA’s Animal Ethics Office. Based on this experience and risk, an alternative technique for administering tiletamine-zolazepam using a pole syringe was developed specifically for this species to reduce the risk of hitting animals in the wrong spot (R Bencini, personal communication 2019). Although using the pole syringe eliminates the risk associated with the use of dart guns, it has a limited range compared to dart guns and still requires the use of a Schedule 8 drug.

Lastly, researchers need to ensure the safe landing of animals that fall from trees. This can be achieved by 2–3 researchers holding a large landing blanket below the animal, as is already the practice with other species (McGregor *et al.* 2016); however, the animal can move quickly before the drug takes effect and fall beyond the banket or become lodged in a tree fork. This occurred on very few occasions (K Yokochi, personal communication 2015), and although it did not result in injuries or death, it remained a cause for concern on animal welfare grounds. The use of a chemical immobilisation regime with tiletamine-zolazepam also requires animals to be kept in captivity for a period of up to 24 hours post-capture, so that their recovery can be monitored prior to release the following night.

There have been attempts to develop a capture method for the ngwayir not involving drey robbing or the use of a chemical immobilisation regime. Wayne *et al.* (2005) compared different survey methods for the ngwayir, one of which entailed using arboreal cage traps. Cage traps were placed on platforms that were attached to trees with self-drilling screws, approximately 1.8 m off the ground. Although this method increased the trap success rate from 0.46 to 3.17% compared to trap placement on the ground, it was not considered effective especially considering the practicalities of installing traps on platforms on trees using self-drilling screws. For a capture method to be widely applicable it needs to be both practical and effective.

Based on the recommendation of a consultant with extensive experience working with the ngwayir (G Harewood, personal communication 2018), Urso (2019) captured the ngwayir using baited wire cage traps strategically positioned above ground on existing tree branches or fences, while another researcher used traps placed on backyard fences to capture the ngwayir in urban areas (B Van Helden, personal communication 2019). Although these researchers had some success in capturing the ngwayir using elevated cage traps, the effectiveness of this capture method has never been investigated.

Given the importance of developing more efficient methods to capture this critically endangered species, we tested whether the placement of cage wire traps on branches or on other raised structures such as fallen logs and fences would increase the capture rate of the ngwayir, compared with conventional ground-level trap placement. This was done by opportunistically analysing data from three different studies, all collected in the same area around Locke Nature Reserve near Busselton, WA, Australia at different times and seasons.

## Materials and methods

### Ethical approval

All the procedures described in this article were approved by the Animal Ethics Committee at The University of Western Australia (RA/3/100/539 and RA/3/100/1213). We conducted our fieldwork following The Australian Code of Practice for the Care and Use of Animals for Scientific Purposes endorsed by the National Health and Medical Research Council of Australia (NHMRC 2004) and followed DBCA’s Standard Operating Procedures (Department of Biodiversity, Conservation and Attractions 2023).

### Study site

**Figure 2.**
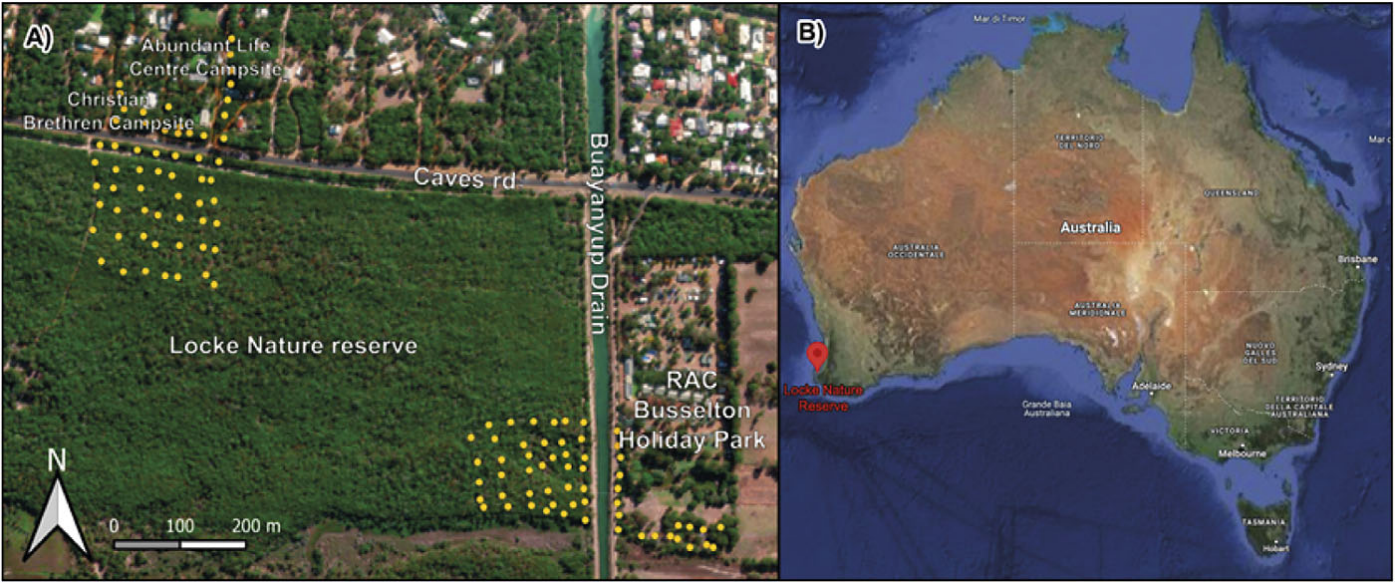
Trap locations for the studies on the ngwayir or western ringtail possum (*Pseudocheirus occidentalis*) conducted between 2010 and 2013 in Locke Nature Reserve and adjacent campsites: the Christian Brethren and the Abundant Life Centre campsites, located across Caves Road and the RAC Busselton Holiday Park located across the Buayanyup Drain (A). The trap locations for the 2019 studies covered the same areas. Locke Nature Reserve and adjacent campsites are located in the south-west of Western Australia (B). (Base map: World Imagery, Sources: Esri, DigitalGlobe, GeoEye, i-cubed, USDA FSA, USGS, AEX, Getmapping, Aerogrid, IGN, IGP, swisstopo, and the GIS User Community).

The ngwayir was captured in 2010–2013 and in 2019 in Locke Nature Reserve and three adjacent campsites, approximately 9 km west of Busselton, where the peppermint tree (*Agonis flexuosa*) woodland supports a high density of the species (Jones *et al.* 1994a, de Tores & Elscot 2010). Locke Nature Reserve covers an area of approximately 200 ha and is managed by the Western Australian Department of Biodiversity, Conservation and Attractions (DBCA). The campsites included the Abundant Life Centre and the Christian Brethren campsites located north of Caves Road and the RAC Busselton Holiday Park east of the Buayanyup Drain (Figure 2).

#### Trapping

We used wire cage traps (450 × 220 × 220 mm [length × width × height], Sheffield Wire Products, Welshpool, WA, Australia) baited with universal bait (a mixture of rolled oats, peanut butter and sardines) and/or pieces of apples or pears. The bait was placed at the back of the traps, which were covered with hessian sacks to provide shelter for trapped animals. Traps were opened in the afternoon and checked at first light, with all traps cleared within 3 h of sunrise as required by DBCA’s Standard Operating Procedures (Department of Biodiversity, Conservation and Attractions 2023).

Between 2010 and 2013, we deployed four trapping grids in Locke Nature Reserve and adjacent campsites. These grids covered an area of approximately 8.25 ha. Traps were placed on the ground with universal bait and pieces of apples for a total of 1,985 trap nights (number of traps × number of nights). Although these grids were originally designed to capture the sympatric common brushtail possum (koomal; *Trichosurus vulpecula*), they intersected the home ranges of resident ngwayirs, that therefore had the opportunity to enter the traps (Yokochi *et al.* 2015).

**Figure 3.**
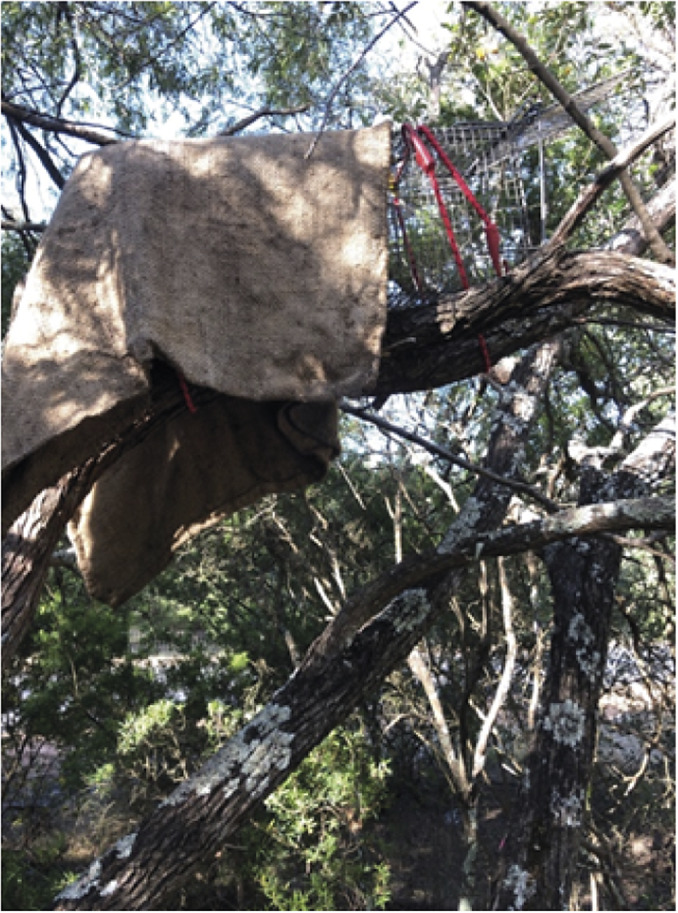
One of the wire cage traps used for the capture of the ngwayir (western ringtail possum; *Pseudocheirus occidentalis*). The trap was secured to the branch using octopus straps. The bait was placed at the back of the trap, which was covered with hessian sacks to provide cover for trapped animals (Photograph: R Bencini).

In March–May and November 2019, the wire cage traps were strategically placed upon horizontal tree branches and fallen trees in Locke Nature Reserve and, in campsites, also on fences, approximately 1.5–2.0 m above the ground for a total of 694 trap nights. We used universal bait without fruit for a total of 106 trap nights and fruit (pieces of apples and pears) for a total of 588 trap nights. Traps were secured using octopus straps and occasionally propped with locally sourced pieces of wood, usually placed below the trap’s entrance, to ensure stability and that the trap doors operated correctly (Figure 3). When a trap was found occupied, it was carefully removed from the branch and lowered to the ground, where the captured animal could either be coaxed into a dark calico animal handling bag or released on-site if it was a bycatch.

#### Statistical analysis

Due to the generally low capture rate of the ngwayir, we converted our data to presence-absence data for each trap night (N = 2,679). A Chi-squared test of independence was conducted using data from all on-ground traps and elevated traps baited with fruit (n = 1,985 and 588 for on-ground and elevated, respectively) to assess whether placing traps above ground baited with fruit resulted in a higher capture rate than the conventional trapping (i.e. placing traps on the ground with universal bait and fruit). A Generalized Linear Mixed Model (GLMM) with binomial distribution was conducted only with data from spring and autumn to assess the influence of trap placement on trap success, while adjusting for the potential impact of the season (i.e. season was set as a random effect; n = 1,762); an adequate dispersion was confirmed for this model. We finally conducted another Chi-squared test of independence to examine if the type of bait influenced the trap success rate in elevated traps (n = 106 and 588 for universal bait and fruit, respectively).

Both Chi-squared tests were conducted using the IBM SPSS® software (Version 28.0, Armonk, NY, USA), while the GLMM was conducted using ‘lme4’ package (Bates *et al.* 2015) in R statistical software (R Core Team 2020).

## Results

### Trapping success rates

In traps deployed on the ground between 2010 and 2013, we captured ten ngwayirs out of 1,985 trap nights (trap success rate of 0.5%); of those ten individuals, five (50%) were re-captured at least once. We also captured 156 quendas (*Isoodon obesulus fusciventer*; 7.9% trap success rate), 99 common brushtail possums (5%), 63 black rats (*Rattus rattus*; 3.2%), 32 bobtail skinks (*Tiliqua rugosa rugosa*; 1.6%), 23 King’s skinks (*Egernia kingii*; 1.2%), 16 house mice (*Mus musculus*; 0.8%), three grey fantails (*Rhipidura albiscapa preissi*; 0.2%), two splendid fairy-wrens (*Malurus splendens*), one European rabbit (*Oryctolagus cuniculus*), one Australian raven (*Corvus coronoides*), seven other non-identified birds, and one non-identified lizard.

In elevated traps deployed in autumn 2019, we captured 27 ngwayirs over 394 trap nights (trap success rate of 6.8%). Nine (56.2%) of these individuals were re-captured. In elevated traps deployed in spring 2019, we captured 55 ngwayirs out of 300 trap nights (trap success rate of 18.3%), with 24 individuals (43.6%) being re-captured (Table 1). During the 2019 elevated trap surveys, we also captured eleven common brushtail possums (1.6% trap success rate), 12 King’s skinks (1.7%) and five black rats (0.7%).

**Table 1.** Study period, trap placement, number of trap nights, number of ngwayir (western ringtail possum; *Pseudocheirus occidentalis*) captured, and trap success rates in three surveys at Locke Nature Reserve and surrounding campsites near Busselton, WA, Australia

Study period and location	Trap placement	Season	Number of trap nights	Number of western ringtail possums trapped	Trap success rate (%)
2010–2013 Locke NR and Campsites	On ground	Autumn, Winter, Spring, Summer	1,985	10	0.5
March–May 2019 Locke NR and Campsites	Elevated	Autumn	394	27	6.8
November 2019 Locke NR only	Elevated	Spring	300	55	18.3

The Chi-squared test with all data indicated that using elevated traps baited with fruit resulted in higher trap success rates for the ngwayir compared with conventional trapping on the ground (n = 2,573, χ^2^ = 192.25; df = 1; *P* < 0.001; odds ratio = 30). The GLMM with spring and autumn data also confirmed that trap success rate was greater in elevated traps than in conventional traps when the model was adjusted for the season (n = 1,762, coefficient = –3.3912; *P* < 0.001). The second Chi-squared test conducted between the elevated traps baited with universal bait and the elevated traps baited with fruit showed that baiting elevated traps with fruit increased the trap success rate (n = 694, χ^2^ = 4.643; df = 1; *P* = 0.03).

## Discussion

While necessary in gaining essential information for conservation, live capturing of wild animals impacts their welfare (McMahon *et al.* 2012), and wildlife scientists seek to reduce these impacts while ensuring the effectiveness of the capture method. Current capture methods for the ngwayir use a chemical immobilisation regime, which carries animal welfare risks and sometimes, as is the case with tiletamine-zolazepam, requires holding of the captured animals in captivity for a variable period of time until the animals recover (Mayberry *et al.* 2014). These methods are also time and labour intensive for conservationists (Clarke *et al.* 2013). Given this, we examined the effectiveness of a novel, practical trapping method and found that the elevated traps increased the trapping success rate of the ngwayir compared with conventional traps placed on the ground while eliminating the welfare risks and technical challenges that were associated with the use of dart guns and a chemical immobilisation regime.

The capture rate of the ngwayir increased from 0.5% to 6.8% (autumn) and 18.3% (spring) when traps were placed upon tree branches, fallen logs or fences, compared with conventional placement of traps on the ground. A GLMM also showed a consistent trend when season was considered, demonstrating that placing traps above ground is more effective when capturing this threatened possum species. Wayne *et al.* (2005) found that the trap success rate increased from 0.46% to 3.17% when the traps were placed on platforms attached to trees; however, their technique did not lead to a broader uptake due to the small increase in trap success rate and difficulties with utilising platforms attached with self-drilling screws on trees to secure the traps. Our method is simple and more likely to be adopted since we rely upon existing horizontal branches, fallen logs and fences that are suitable for trap placement, and use elasticised octopus straps that are widely available to firmly secure the traps.

Since we only had data for the autumn and spring of 2019, we evaluated the effect of seasonality as a random effect in the model. It would be necessary to collect data for more seasons to better interpret the effect of season on the efficiency of this trapping method. The higher trap success rate in spring could be due to the placement of some of the traps in campsites during the autumn of 2019, while in spring 2019 traps were only deployed within Locke Nature Reserve, which has one of the highest recorded densities of ngwayirs (de Tores & Elscot 2010).

In general, an increase in trap success rate could be due to an increase in population density; however, this is unlikely in our study location. The density of the ngwayir in Locke Nature Reserve was reported to be 5.84 individuals per ha in 2010 based on a distance sampling study (de Tores & Elscot 2010). This density had almost certainly decreased by 2019, given the predicted sharp decline for this population based on a population viability analysis (Yokochi 2015). Indeed, using the same distance sampling protocol as de Tores and Elscot (2010), Harring-Harris (2014) reported that the density in Locke Nature Reserve had dropped to 1.03–1.51 individuals per ha by 2014, matching the rate of decline predicted by Yokochi (2015). In a recent survey at the same location, a consultant found a density of 3.05 individuals per ha (Teale & Potts 2020), indicating that the density had increased between 2014 and 2019, but not to the levels reported by de Tores and Elscot in 2010. Given this declining trend in the population density of the ngwayir in our study location from 2010 to 2019, the increase in capture rate in our study is unlikely to be due to changes in the population density, but more likely due to the change in the placement of the traps. There is still a possibility that other variables influenced trap success, such as food availability. An experiment with traps set on the ground and above ground at the same time, could provide more accurate data and confirm the greater effectiveness of elevated traps compared to ground traps when trapping ngwayir. However, such a study would have implications both for animal welfare and resources.

The influence of bait types alone could not be rigorously tested in this study due to the lack of overlap in the different bait types for both placements of the traps in our surveys; however, when we analysed data from elevated traps only, using fruit increased the capture rate of the ngwayir. Wayne *et al.* (2005) reported that the bait type did not influence capture rates of the ngwayir; however, they compared universal bait, rose oil and eucalyptus oil, not fruits. Given the folivorous nature of the ngwayir, the wide availability of fruit, and our success in capturing them using pieces of fruit, we recommend using fruit as bait when trapping these possums.

Our data also suggest that the ngwayir shows neither neophobia nor the development of trap shyness towards wire cage traps. Individuals entered the traps likely attracted by the bait, and 43.6% to 50% of the individuals trapped were re-captured, suggesting that these possums may even become trap happy, contrary to what was suggested by Wayne *et al.* (2005). This is advantageous because studies of wildlife populations often require multiple captures of the same individuals, for example to monitor their health or retrieve tracking devices. Indeed, one of the concerns with a species that is difficult to capture is with the recovery of telemetry devices (e.g. radio-transmitter collars) that have been deployed on the animals. In the past, attempts to recapture ngwayirs to retrieve radio transmitter collars proved to be difficult because the collared possum had to be close enough and in a suitable position on a tree to be reached using a dart gun or a pole syringe. With elevated traps, we have been able to efficiently recapture radio-collared possums and retrieve their collars, which results in improved welfare for the animals that do not have to spend the rest of their lives wearing collars.

Using elevated traps is likely to improve the welfare of the captured ngwayir. To date, effective capturing of the ngwayir required the use of a dart gun or pole syringe to administer a chemical immobilisation regime. To prevent the animal from falling to the ground, a landing blanket was used to cushion the fall, but this technique is not perfect, with some animals falling beyond the landing blanket or becoming lodged in tree forks (Nilsen *et al.* 2009; McGregor *et al.* 2016; Hampton *et al.* 2022). The use of a dart gun involves additional difficulties because, even with thorough training and developed marksmanship, hitting a small target (rump) on a small animal at a distance of up to 10 m can be challenging if the animal moves suddenly and unexpectedly. Incidents of injuries due to captures by a dart gun are rare (two in 329 captures, unpublished data) but present, nonetheless. These problems can be overcome by using elevated traps as a capture method.

In addition, elevated trapping is also more practical than a chemical immobilisation regime of animals using a dart gun or a pole syringe because it requires less specialist training for personnel by removing the need for firearms or drugs; this improvement will, in turn, likely lead to greater efficiency in surveying and monitoring this critically endangered species.

Elevated traps also reduced the capture rates of non-target species (e.g. the quenda) compared to traps on the ground. Target specificity is one of the main welfare considerations when conducting live capture, especially by trapping (Allen *et al.* 2022), and this novel method likely improves the welfare of other, non-target species in the survey area.

### Animal welfare implications

We have developed and tested a novel methodology for capturing the ngwayir that dramatically increases trap success compared to the conventional placement of traps on the ground. This method presents an effective alternative to conventional and potentially risky methods requiring a chemical immobilisation regime using a dart gun or a pole syringe. The increase in trap success rates from this method has the potential to support conservation actions for this critically endangered species by improving the welfare of captured animals and efficiencies for researchers attempting to study the species to address knowledge gaps. This widely applicable trapping approach also has the potential to be adopted for other similar arboreal species that show low trap success rates using conventional trapping methods.
